# The Time of Blood Collection Does Not Alter the Composition of Leucocyte-Poor Platelet-Rich Plasma: A Quantitative Analysis of Platelets and Key Regenerative Proteins

**DOI:** 10.3390/cimb47100788

**Published:** 2025-09-23

**Authors:** Hadrian Platzer, Alena Bork, Malte Wellbrock, Axel Horsch, Ghazal Pourbozorg, Simone Gantz, Reza Sorbi, Sébastien Hagmann, Yannic Bangert, Babak Moradi

**Affiliations:** 1 Department of Orthopedics and Trauma Surgery, University Medical Center Schleswig-Holstein, Campus Kiel, Arnold-Heller-Straße 3, 24105 Kiel, Germany; hadrianmarius.platzer@uksh.de (H.P.); reza.sorbi@uksh.de (R.S.); 2 Orthopedic Research Center, Kiel University, Michaelisstr. 5, 24105 Kiel, Germany; malte.wellbrock@gmx.de (M.W.); ghazal.pourbozorg@uksh.de (G.P.); simone.gantz@uksh.de (S.G.); 3 Department of Orthopedics, Heidelberg University Hospital, Schlierbacher Landstraße 200a, 69118 Heidelberg, Germany; alena.bork@stud.uni-heidelberg.de (A.B.); axel.horsch@med.uni-heidelberg.de (A.H.); sebastien.hagmann@atos.de (S.H.); yannic.bangert@med.uni-heidelberg.de (Y.B.)

**Keywords:** leucocyte-poor platelet-rich plasma, PRP, regenerative proteins, growth factors, cytokines, blood collection time

## Abstract

Platelet-rich plasma (PRP) is increasingly used in orthopedics, with its regenerative potential attributed to platelet-derived cytokines and growth factors. However, variability in PRP composition hampers standardization and reproducibility, contributing to inconsistent outcomes. Since platelet counts in whole blood follow circadian rhythms, we investigated whether blood collection time affects PRP composition. Venous blood was collected from 25 healthy individuals at 8:00 a.m., 12:00 p.m., and 4:00 p.m. Whole blood was analyzed, and leukocyte-poor PRP (LP-PRP) was prepared for quantification of erythrocytes, leukocytes, and platelets. Platelets were lysed by freeze–thaw cycles, and protein levels of PDGF-BB, IGF1, HGF, IL6, and IL10 were measured via ELISA. Whole blood exhibited diurnal variation in platelet counts. In contrast, LP-PRP was consistently depleted of leukocytes and erythrocytes and showed stable platelet enrichment (PRP/whole blood ratio 2.1 ± 0.3). Protein concentrations in LP-PRP did not differ across collection times. Despite marked interindividual variability, no time-dependent differences were observed in platelet or protein composition. These results demonstrate that LP-PRP is temporally stable, independent of blood sampling time. This robustness supports flexible clinical use and contributes to efforts toward PRP standardization in therapeutic practice.

## 1. Introduction

Platelet-rich plasma (PRP) has emerged as a promising therapeutic option in orthopedic and sports medicine particularly for the management of musculoskeletal disorders such as osteoarthritis, muscle injuries, and cartilage repair [1,2,3,4,5]. The therapeutic effects of PRP are primarily attributed to cytokines and growth factors promoting tissue healing and modulating inflammation [4,6,7]. PRP contains a variety of proteins, with the exact number varying depending on the source and PRP preparation, with estimations comprising up to 600 different proteins, including those associated with blood clotting, healing, and immune response [8,9,10,11]. However, outcomes across studies remain inconsistent, and the mechanisms by which PRP exerts its therapeutic effects, especially in the context of orthopedic conditions like osteoarthritis, are not well understood.

A primary challenge in PRP research is the variability in its composition, which has yet to be thoroughly investigated. This variability complicates efforts to standardize PRP protocols and evaluate its efficacy across studies and clinical settings. PRP preparation is influenced by multiple factors, including centrifugation techniques, activation methods, and the presence or absence of anticoagulants, all contributing to PRP’s heterogeneity [12,13]. Moreover, individual patient-specific factors further impact PRP composition [14,15]. For example, age seems to significantly influence levels of growth factors such as insulin-like growth factor 1 (IGF1) and transforming growth factor-beta (TGF-β) in PRP [14,15,16]. Additionally, circadian variations in platelet biology raise questions about the optimal timing for blood collection in PRP preparation. Studies analyzing whole blood demonstrated that both platelet count and function exhibit diurnal fluctuations, with peak platelet counts occurring in the afternoon [17,18]. However, peak platelet activity does not appear to coincide with peak counts. Platelet aggregation has been shown to reach its maximum in the morning [17,19,20]. Such findings suggest that circadian fluctuations in platelet count and activity may also influence the composition of PRP. Although the timing of PRP preparation may have important clinical implications, only a single study of a small cohort of ten healthy individuals analyzed the PRP composition depending on blood collection time and showed stable platelet counts, TGF-β and PDGF-BB levels throughout the day [21]. With this isolated study, the dependence of the PRP composition and its activity on the time of blood collection has obviously only been insufficiently studied at present.

Our study aimed to clarify the influence of blood collection time on the concentration of platelets in PRP and the consistency of both PRP growth and cytokine levels. In this study we analyzed leukocyte-poor PRP (LP-PRP) to reduce variability introduced by leukocyte-derived components and to ensure a more standardized and homogeneous product, allowing a clearer evaluation of potential circadian influences on platelets and platelet-derived proteins.

The proteins in PRP investigated in this study were chosen based on their roles in tissue repair and inflammation and were identified as critical mediators in immunomodulation and recovery of orthopedic conditions. Interleukin-6 (IL6), traditionally classified as a pro-inflammatory cytokine, exemplifies a dual role in tissue response and repair, mediating both catabolic and protective processes [22,23]. Hepatocyte Growth Factor (HGF) regulates epithelial and endothelial cell motility and proliferation. HGF facilitates epithelial repair and promotes neovascularization during wound healing, underscoring its significance in reparative mechanisms [4]. Platelet-derived growth factor (PDGF-BB) enhances chemotaxis and mitogenesis in fibroblasts and smooth muscle cells, regulating collagenase secretion and promoting collagen synthesis, key steps in tissue repair [4]. Insulin-like growth factor-1 (IGF1) serves as a chemotactic factor for fibroblasts, stimulating their migration and protein synthesis, while also promoting osteoblast proliferation and differentiation [4,24,25]. Finally, Interleukin-10 (IL10) was selected for its anti-inflammatory properties and its ability to modulate the immune response during tissue repair [26,27].

Specifically, this study investigated in a well-characterized cohort whether the timing of whole blood collection (8:00 a.m., 12:00 p.m., and 4:00 p.m.) affects platelets as well as levels of aforementioned growth factors and cytokines in LP-PRP. Through this, we aimed to determine whether the time of blood collection represents a relevant source of variability in PRP composition that may affect its clinical applicability.

## 2. Materials and Methods

### 2.1. Study Population

A total of 25 healthy participants (12 males, 13 females) were enrolled in this study, with a mean age of 31.4 ± 3.5 years and a mean BMI of 22.8 ± 2.3 kg/m^2^ (Table 1). 

None of the participants had a history of malignancy, chemotherapy or hematologic disorders. In addition, exclusion criteria included acute musculoskeletal injury, surgery within the past six months, or the use of disease-modifying antirheumatic drugs (DMARDs) or corticosteroids within the previous three months prior to study initiation. The use of non-steroidal anti-inflammatory drugs (NSAIDs) or paracetamol within six weeks, or acetylsalicylic acid within two weeks preceding study participation, also led to study exclusion. Furthermore, no participant showed clinical signs of acute or chronic inflammation, as assessed by physical examination and whole blood analysis. The study was approved by the ethics committee of the University of Heidelberg (S-631/2021), and written informed consent was obtained from all participants prior to enrollment.

### 2.2. Sample Collection

Blood was taken from the peripheral arm vein at three different time points (8 a.m., 12 p.m. and 4 p.m.). EDTA tubes (S-Monovette^®^ EDTA K3E, 2.7 mL; Sarstedt, Nümbrecht, Germany) were used to collect blood for subsequent haematological examination. Blood was also collected using a double-syringe system (Arthrex ACP system; Arthrex, Naples, FL, USA) for the preparation of PRP. All samples were immediately further processed.

### 2.3. PRP Preparation

After blood collection into the ACP double-syringe system, PRP was prepared by centrifugation at 1500 rpm for 5 min (centrifuge used: Horizon 24-AH, Drucker Diagnostics, Port Matilda, PA, USA) according to the manufacturer’s instructions (see protocol Arthrex Naples, FL, USA, 2023). PRP samples were frozen at −80 °C (Dometic UF 755 GG Ultra Low Temperature Freezer; Dometic, Solna, Sweden) in 1.5 mL Eppendorf tubes (Eppendorf AG, Hamburg, Germany) within the first 30 min after PRP preparation. Two freeze–thaw cycles were performed to release PRP proteins from platelets in vitro as previously described [28,29]. This approach ensured uniform platelet disruption and protein release across all samples, while minimizing donor-dependent variability and avoiding potential confounding effects of exogenous agents. Immediately before the first thawing process, coagulation of PRP samples was prevented by adding 20 µL of unfractionated heparin-natrium (25.000 I.U./5 mL; LEO PharmaA/S, Ballerup, Denmark) per 1 mL of sample. The samples were divided into aliquot portions according to the subsequent analysis and again frozen until protein analysis. All samples were meticulously treated in the same way until protein analysis.

### 2.4. Hematological Analysis

The cell concentrations of platelets, erythrocytes and leucocytes were determined from all samples, whole blood and PRP, using an automated haematology analyzer Sysmex XN-1000 (Sysmex Corporation, Kobe, Japan) to firstly ensure that whole blood samples were within normal cell concentrations and showed no signs of infection and secondly to confirm that the platelet enrichment in PRP samples was successful.

### 2.5. Protein Analysis

PRP samples were thawed to room temperature and centrifuged through a filter plate (AcroPrep Advance 96 Well Plates, 350 µL, 3 μm glass fiber/0,2 μm Supor Short Tip Natural PP Base Membrane; Pall Corporation, Port Washington, NY, USA) for 10 min at 1400× *g* prior to analysis to remove cell debris from the lysed platelets. The concentrations of IL6, IGF1, HGF, PDGF-BB were then measured by enzyme-linked immunosorbent assay (ELISA) according to the manufacturer’s protocol (R&D Systems, Minneapolis, MN, USA, lot number IGF1: P310291; lot number IL6: P320482//Sigma Aldrich, St. Louis, MI, USA; lot number PDGF-BB: 0307J180; lot number HGF: 0308K0201). Standard curves prepared from recombinant proteins provided with each kit served as positive controls (run in duplicate), while two blank wells containing assay buffer and reagents but no sample were included as negative controls, in accordance with the assay protocols. Subsequently, samples were immediately read at 450 nm using the Microplate photometer from Autobio Labtec Instruments Co. (Software AUTOsoft, version 2.6.9; Zhengzhou, China).

### 2.6. Statistical Analysis

Data are presented as mean (±standard deviation) or median with interquartile range (IQR) for continuous variables, and as count (percentage) for categorical variables, as indicated. The Shapiro–Wilk test was used to test for normality of the continuous variables.

To analyze significant differences in the variability of components of blood and protein concentrations throughout the day, the two-factor non-parametric repeated measurements analysis of variance (ANOVA) for ranks according to Friedman was conducted. For each protein, adjustments for multiple comparisons were made using the Bonferroni correction, if necessary, to control for the risk of Type I errors across the analysis.

The coefficient of variation (CV), defined as the ratio of the standard deviation to the mean (in percentage), was used to describe interindividual variability in protein concentrations at each blood collection time point (8 a.m., 12 p.m., 4 p.m.). This enabled comparison of variability across proteins independent of absolute concentration levels. To test whether variability differed between time points, Levene’s test was applied to assess the homogeneity of variances.

Tests were two-tailed, and statistical significance was defined as a *p* value < 0.05 for estimate from Friedman ANOVA for ranks. Statistical analysis was performed using SPSS version 29.0.2.0 (IBM Corp., Armonk, NY, USA). Graphs were generated using GraphPad Prism version 10 (GraphPad Software Inc., La Jolla, CA, USA).

## 3. Results

### 3.1. Cell Profile in Whole Blood and LP-PRP Across Blood Collection Times

Concentrations of whole-blood cell populations of all samples were within physiological range. In whole blood, total leukocyte concentrations at 12 p.m. (*p* = 0.009) and 4 p.m. (*p* < 0.001) were significantly elevated compared to the 8 a.m. samples. In contrast, monocyte levels were significantly decreased at 12 p.m. (*p* < 0.001) and 4 p.m. (*p* = 0.022), as were eosinophil concentrations at 12 p.m. (*p* = 0.002) and 4 p.m. (*p* = 0.040) compared to the 8 a.m. time point (Table 2).

Erythrocytes (mean 0.03 ± 0.04 × 10^3^/nL) and leukocytes (mean 0.14 ± 0.30/nL) in PRP were nearly depleted and not further analyzed. The mean PRP-to-whole-blood platelet ratio was 2.1 ± 0.3 and remained consistent across different time points (Table 2).

**Figure 1:**
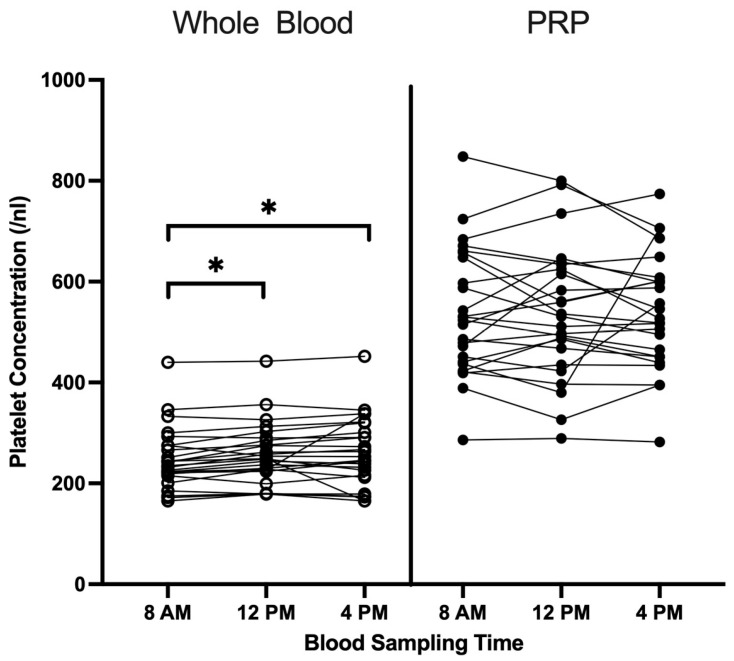
Platelet concentrations in whole blood and LP-PRP depending on blood collection time (8 a.m., 12 p.m. and 4 p.m.) are shown (*n* = 25). Raw data are plotted. * = *p* < 0.05.

Whole-blood platelet concentrations were significantly higher at 12 p.m. (*p* = 0.022) and 4 p.m. (*p* = 0.027) compared to samples collected at 8 a.m. However, PRP samples showed no significant differences in platelet concentrations across blood collection time points (Figure 1).

### 3.2. Cytokine and Growth-Factor Analysis in LP-PRP Across Blood Collection Times

**Figure 2:**
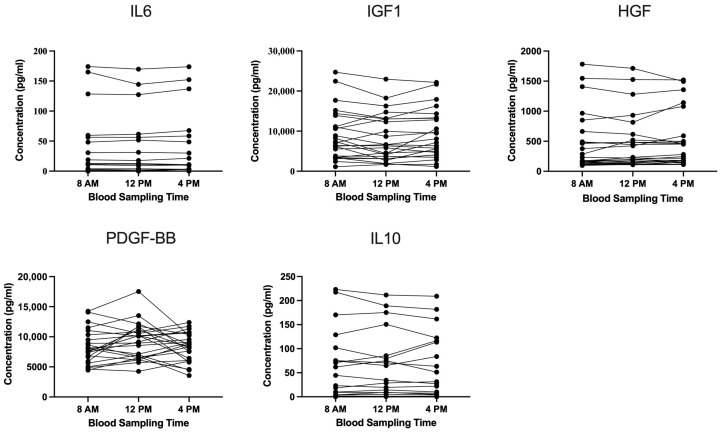
Protein concentrations in LP-PRP at different blood collection times. The concentrations of IL6 (*n* = 25), IGF1 (*n* = 25), HGF (*n* = 23), PDGF-BB (*n* = 24) and IL10 (*n* = 19) in PRP at blood collection times 8 a.m., 12 p.m. and 4 p.m. are shown in pg/mL. The analysis was performed using an Enzyme-Linked Immunosorbent Assay (ELISA). Raw data are plotted.

Substantial interindividual variability in cytokine and growth factor levels was observed. Despite strict exclusion criteria and a homogeneous, well-characterized study population, the underlying sources of this variability was not identified based on the available data. Importantly, Friedman ANOVA revealed no significant differences in PDGF-BB, HGF, IGF1, IL6, or IL10 concentrations across blood collection time points (Figure 2). 

In three participants, IL6, HGF, and IL10 levels were consistently elevated. One individual showed uniformly high levels of all three markers, while another exhibited elevated concentration of both IL10 and HGF. No time-dependent differences were observed in these outliers, and no clinical or demographic parameters were identified that could explain their consistently elevated levels.

### 3.3. Analysis of Data Variability in LP-PRP Based on Blood Collection Times

Table 3 presents the coefficient of variation (CV) for each protein measured at 8 a.m., 12 p.m., and 4 p.m. The CV values reflect the degree of interindividual variability in protein concentrations within each respective time point. 

As expected, the magnitude of variability differed between proteins: PDGF-BB showed relatively low variability, IGF1 moderate variability, and HGF higher variability, while IL10 and IL6 displayed the highest fluctuations. However, the degree of variability for each protein remained stable across time points, and no diurnal pattern was observed.

Consistently, Levene’s test revealed no significant differences in variance across the three time points for any of the analyzed proteins, further supporting the temporal stability of LP-PRP composition over the course of the day.

## 4. Discussion

Despite several studies reporting promising outcomes with PRP [1,2,3,4,5], the overall evidence for its consistent benefit in orthopedics remains inconclusive due to heterogeneous results across the literature. Consequently, major international guidelines currently do not recommend PRP for osteoarthritis, even though it is one of the most common orthopedic indications for its use [30]. A key factor likely contributing to these inconsistent findings is the variability in PRP composition, which remains insufficiently understood [14]. Since platelet counts and activity in whole blood follow a circadian rhythm [17,18,19,20], we hypothesized that the time of blood collection may influence PRP composition and, by extension, its therapeutic efficacy. Our study therefore aimed to investigate whether the timing of blood collection affects the cellular or molecular profile of PRP—a question of considerable clinical relevance. We selected the time points 8:00 a.m., 12:00 p.m., and 4:00 p.m. to reflect clinically relevant intervals within routine medical practice.

Quantification of platelets, erythrocytes, and leukocytes in whole blood confirmed that all values were within the normal physiological range. LP-PRP preparation was successful, achieving a mean platelet enrichment ratio of 2.1 compared to whole blood, with near-complete leukocyte and erythrocyte depletion. Given the use of LP-PRP, our findings apply specifically to preparations devoid of leukocytes. Caution is warranted when extrapolating these results to leukocyte-rich PRP formulations, particularly in relation to cytokine content, as immune cells are known to contribute significantly to such protein levels.

As expected, platelet counts in whole blood exhibited significant circadian fluctuations, with higher levels observed at 12:00 p.m. and 4:00 p.m. compared to 8:00 a.m. This aligns with previous findings demonstrating a peak in circulating platelet levels during the afternoon [17,18]. However, these circadian fluctuations were not reflected in the LP-PRP samples, where platelet concentrations remained stable across all time points. This suggests that while the timing of blood sampling seems to influence platelet counts in whole blood, it does not affect PRP, confirming earlier observations [21].

Given that PRP protein levels might be influenced by circadian rhythms independently of platelet counts, we additionally analyzed five key proteins—PDGF-BB, IGF1, HGF, IL6, and IL10—all of which are essential mediators of immune regulation and play a critical role in tissue regeneration and recovery from musculoskeletal disorders [4,23,24,26]. To induce the release of platelet-derived cytokines and growth factors, we applied two freeze–thaw cycles, a well-established method for in vitro protein liberation from platelets [28,29]. In our study no significant differences in protein concentrations across blood collection times were detected. The differences in fluctuations show that each protein follows a distinct pattern of variation, yet all remain within physiologically normal ranges. Fluctuations remained stable throughout the day, and no specific time point showed a pronounced increase in variability, as confirmed by the coefficient of variation values across time.

However, although PRP composition appeared temporally stable, considerable interindividual variability in cytokine and growth factor concentrations was evident. This was particularly pronounced for HGF, IL10, and IL6. In this study, a well-characterized and homogeneous cohort of healthy individuals was included, and extensive exclusion criteria were applied to minimize confounding by comorbidities or medication use. Nevertheless, substantial variability persisted across participants, suggesting that other unaccounted factors may have influenced PRP protein composition. This observed variability in PRP’s bioactive components—likely affecting its efficacy—highlights the need for further research on determinants of PRP composition.

Taken together, our results suggest that LP-PRP’s therapeutic components, at least key proteins and cells analyzed in this study, remain stable throughout the day, which is of enormous practical relevance for clinical applications, where flexibility in blood collection timing is highly beneficial. The absence of time-of-day-dependent variability enhances logistical flexibility and supports consistent application independent of appointment scheduling.

Our results should be interpreted considering certain limitations. While the sample size of 25 participants may be considered modest, the repeated-measures design, strict inclusion criteria, and standardized methodology enhance the reliability and internal validity of our findings. As an in vitro study, our results may not fully capture the complexity of in vivo conditions, where additional physiological factors could influence PRP composition and therapeutic efficacy. Moreover, the present study relied on ELISA as the sole detection method. While this approach provided sensitive and quantitative measurements of predefined proteins, complementary techniques such as Western blotting could further validate and extend our findings and should be considered in future studies. Lastly, although we focused on key cytokines and growth factors, other biologically active molecules were not assessed, potentially overlooking further circadian influences.

## 5. Conclusions

This study demonstrates that the composition of LP-PRP—with both cellular and protein components analyzed—remains stable across different blood collection times. Our findings support the flexible and reliable use of LP-PRP in clinical practice, regardless of appointment timing. However, the substantial interindividual variability observed—particularly in cytokine levels—highlights the need for further evaluation of the underlying heterogeneity of PRP. A more comprehensive understanding of PRP composition and its biological variability is essential to optimize its therapeutic potential in regenerative and orthopedic medicine. Furthermore, the standardization of PRP preparation protocols is key to improving study comparability and strengthening the evidence base for its clinical application.

## Figures and Tables

**Table 1:** Demographic and clinical parameters of the study population.

	Total Study Population
Number of patients, *n*	25
Sex, *n*; %	
Female	13; 52%
Male	12; 48%
Age in years, mean ± SD; IQR	31.44 ± 3.54; 5.00
BMI ^1^ (kg/m^2^), mean ± SD; IQR	22.81 ± 2.31; 3.24
Blood cell concentration (whole blood), mean ± SD; IQR	
Leucocytes (/nL)	6.18 ± 1.34; 2.13
Erythrocytes (×10^3^/nL)	4.85± 0.35; 0.48
Platelets (/nL)	255.60 ± 61.72; 68.67

^1^ BMI = Body Mass Index.

**Table 2:** Cell analysis in whole blood and LP-PRP at different blood collection times.

	Time	8 a.m.		12 p.m.		4 p.m.		*p*-Value
		mean	SD	mean	SD	mean	SD	
Whole blood ^1^	Platelets (/nL)	248.08	60.97	257.72	60.70	261.00	68.98	0.008
	Erythrocytes (×103/nL)	4.87	0.39	4.83	0.37	4.84	0.35	0.089
	Leukocytes (/nL)	5.49	1.29	6.30	1.50	6.76	1.57	<0.001
	Lymphocytes (%)	33.10	7.14	32.72	7.65	34.03	7.45	0.468
	Monocytes (%)	5.64	1.40	4.85	1.25	5.21	1.09	<0.001
	Basophils (%)	0.72	0.21	0.67	0.21	0.61	0.22	0.088
	Eosinophils (%)	3.36	1.90	2.69	2.00	2.84	1.99	0.001
	Neutrophils (%)	54.76	7.82	56.82	8.90	55.22	7.52	0.326
PRP ^1^	Platelets (/nL)	537.04	126.27	538.00	130.65	539.84	114.95	0.619
PRP-to-whole blood platelet ratio ^1^		2.10	0.38	2.10	0.32	2.10	0.32	0.432

^1^ Mean values and standard deviations (SD) of cell concentrations and corresponding *p*-values from comparative analyses between blood collection times (8 a.m., 12 p.m. and 4 p.m.) are shown. Leukocyte subpopulations are presented as percentages (%) of the total leukocyte population.

**Table 3:** Coefficient of Variation (CV, %) reflecting interindividual variability of PRP protein concentrations measured at 8 a.m., 12 p.m., and 4 p.m.

	IL6	IGF1	HGF	PDGF-BB	IL10
8 a.m.	177.82	69.24	102.98	33.67	111.15
12 p.m.	173.53	71.07	96.06	32.25	107.51
4 p.m.	173.69	65.78	90.59	28.62	106.30

## Data Availability

The data presented in this study are available from the corresponding author upon reasonable request and subject to institutional and ethical approval.

## Abbreviations

The following abbreviations are used in this manuscript:

ANOVA	Analysis of Variance
CV	Coefficient of Variation
DMARD	Disease-Modifying Anti-Rheumatic Drug
EDTA	Ethylenediaminetetraacetic Acid
ELISA	Enzyme-Linked Immunosorbent Assay
HGF	Hepatocyte Growth Factor
IGF	Insulin-like Growth Factor
IL	Interleukin
IQR	Interquartile Range
LP-PRP	Leukocyte-Poor Platelet-Rich Plasma
mL	milliliter
nL	nanoliter
NSAID	Non-Steroidal Anti-Inflammatory Drug
OA	Osteoarthritis
PDGF	Platelet-Derived Growth Factor
pg	picogram
PRP	Platelet-Rich Plasma
SD	Standard Deviation
SPSS	Statistical Package for the Social Sciences
TGF	Transforming Growth Factor
